# A novel FadL family outer membrane transporter is involved in the uptake of polycyclic aromatic hydrocarbons

**DOI:** 10.1128/aem.00827-24

**Published:** 2025-01-24

**Authors:** Qiu Meng, Yuxuan Liang, Yinming Xu, Saiyue Li, Haiyan Huang, Yuanyou Xu, Feifei Cao, Jianhua Yin, Tingheng Zhu, Haichun Gao, Zhiliang Yu

**Affiliations:** 1 College of Biotechnology and Bioengineering, Zhejiang University of Technology 630365 , Hangzhou, Zhejiang, China; 2 Institute of Microbiology, College of Life Sciences, Zhejiang University 98445 , Hangzhou, Zhejiang, China; 3 Hangzhou Chuhuan Science and Technology Co., Ltd., Hangzhou, Zhejiang, China; Universidad de los Andes, Bogotá, Colombia

**Keywords:** polycyclic aromatic hydrocarbons, FadL family, trans-membrane transport, biodegradation, *Novosphingobium pentaromativorans*

## Abstract

Gram-negative bacteria play a pivotal role in the bioremediation of persistent organic pollutants, such as polycyclic aromatic hydrocarbons (PAHs). Because the outer membrane (OM) of these bacteria hinders the direct permeation of hydrophobic substances into the cells, trans-OM proteins are required for the uptake of PAHs. However, neither the characteristics of PAH transporters nor the specific transport mechanism has been well interpreted. In this study, we revealed the participation of a novel FadL family transporter, PadL, in the biodegradation of the representative PAH phenanthrene in *Novosphingobium pentaromativorans* US6-1, an efficient PAH-degrading bacterium. PadL facilitates the cross-OM transport of phenanthrene, thus upregulating the expression of the gene *ahdA1e* that is critical to the PAH catabolism. We then showed that hydrophobic amino acid residues in the substrate binding pockets of PadL are essential for the binding of PAHs, such as phenanthrene and benzo[*a*]pyrene. PadL homologs commonly exist in most of the PAH-degrading species from *Sphingomonas* and *Novosphingobium*. The characterization of PadL provided in this study holds significant potential for improving the PAH biodegradation efficiency.

**IMPORTANCE:**

Persistent organic pollutants, including polycyclic aromatic hydrocarbons (PAHs), pose serious threats to human health, and biodegradation has been applied as an efficient strategy for PAH removal. However, due to the high hydrophobicity of PAHs, their uptake is hindered by the bacterial outer membrane, restraining degradation efficiency. The present study reveals the critical roles of a novel FadL family protein (PadL) in the biodegradation of PAHs. PadL specifically transports PAHs such as phenanthrene and benzo[a]pyrene and PadL homologs generally exist in PAH-degrading bacteria of Sphingomonas and Novosphingobium. Our findings fill the knowledge gap in the bacterial trans-membrane uptake process of PAHs and provide a future direction for enhancing the bacterial PAH bioremediation capacity.

## INTRODUCTION

Polycyclic aromatic hydrocarbons (PAHs) are hydrocarbons characterized by two or more fused benzene rings, typically originating from the incomplete combustion or pyrolysis of organic materials (1). With high structural diversity, PAHs such as naphthalene, phenanthrene, anthracene, and benzo[*a*]pyrene are widespread in the environment (2). The molecular architecture of PAHs, defined by concatenated benzene rings and conjugated double bonds, imparts a notable stability and hydrophobic nature, leading to the prominence of PAHs as persistent environmental pollutants (3). PAHs are detrimental to human health, with documented cases of toxicity, mutagenicity, and carcinogenicity (4). Human exposure primarily occurs through inhalation, ingestion, or dermal contact, with the potential to impair functions of the respiratory, circulatory, and nervous systems (5).

Bacteria possess robust metabolic capacities for pollutant detoxification. For the degradation of PAHs, the use of bacteria offers several benefits, such as high degradation rate, low cost, and absence of secondary pollution, and is currently emerging as one of the most promising approaches (3). To date, various PAH-degrading bacteria have been isolated including *Sphingomonas*, *Novosphingobium*, *Acinetobacter*, *Rhodococcus*, and *Pseudomonas* (6, 7). The bacterial breakdown of hydrophobic PAHs can be delineated into three sequential stages: (i) initial adsorption of the compounds by the cell, (ii) uptake of the adsorbed compounds from the cell membrane surface into the cell via trans-membrane transport, and (iii) catabolism of PAHs driven by catabolic enzymes (8, 9). Among them, the trans-membrane transport of PAHs to cross the bacterial cell envelope is a bottleneck step for biodegradation (10, 11), and attempts have been made to improve biodegradation efficiency by enhancing the PAH transport (12).

Most PAH-degrading microorganisms are gram-negative bacteria that possess an outer membrane (OM) structure. The bacterial OM contains a hydrophilic layer on the outer leaflet that is mainly constituted by lipopolysaccharides (LPS), which hinders the passive diffusion of hydrophobic PAHs (13). Thus, the transport of PAHs across the OM requires specific transporters or channel proteins (8, 14, 15). The documented OM proteins known to facilitate the transport of hydrophobic aromatic compounds include the TonB-dependent transporters (TBDTs), the OmpW family proteins, and the FadL family channel proteins (11, 16–20). Despite widespread attention to the bacterial bioremediation of PAH contamination in recent decades, studies on the trans-membrane transport of PAHs are still scarce, and the mechanistic understanding of PAH transporters is nearly absent.

FadL is an OM channel protein composed of a 14-stranded β-barrel functioning in the transport of hydrophobic hydrocarbons (21). FadL from *Escherichia coli* (*Ec*FadL) is the archetype of the FadL family proteins. The primarily function of FadL is to facilitate the cross-membrane transport of long-chain fatty acids (LCFAs) (22). In addition to LCFAs, some FadL analogs were reported to transport other molecules of physiological significance in gram-negative bacteria (20). For example, two FadL family transporters, TodX and CymD, can transport monocyclic aromatic hydrocarbons in *Pseudomonas putida* PpF1 (20). Additionally, a FadL homolog in *Acinetobacter venetianus* RAG-1, AltL, transports long-chain alkanes and fatty acids (23). However, the molecular mechanism by which the FadL family proteins facilitate the uptake of PAHs remains unknown.

*Novosphingobium pentaromativorans* US6-1, a facultative anaerobic *α*-proteobacterium, was previously identified as the first bacterium capable of degrading PAHs with high molecular weight (24). In this study, we reported that PadL of *N. pentaromativorans* US6-1 (*Np*PadL), a transporter belonging to the FadL family, facilitated the assimilation of PAHs with a high substrate specificity. The substrates of PadL include phenanthrene and benzo[*a*]pyrene. By applying molecular docking simulation and site-directed mutagenesis, we determined that the hydrophobic pocket of PadL is crucial for its PAH transport activity. Evolutionary analyzes showed that PadL homologs are widely distributed in PAH-degrading bacteria of the genera *Sphingomonas* and *Novosphingobium*, but the sequence similarity between PadL and well-studied FadL family proteins such as *Ec*FadL or TodX is rather low (< 30%). This sequence peculiarity suggests that *Np*PadL represents a novel FadL subfamily that is specifically for the substrate analogs of PAHs. Our findings expand the knowledge of the functional diversity among FadL family transporters and provide insights into cross-membrane transport, the long-ignored rate-limiting step in the degradation of hydrophobic substances such as PAHs. This work also provides a perspective for constructing effective microorganisms for the bioremediation of PAH contamination.

## RESULTS

### PadL is crucial for the biodegradation of phenanthrene in *N. pentaromativorans* US6-1

**Fig 1:**
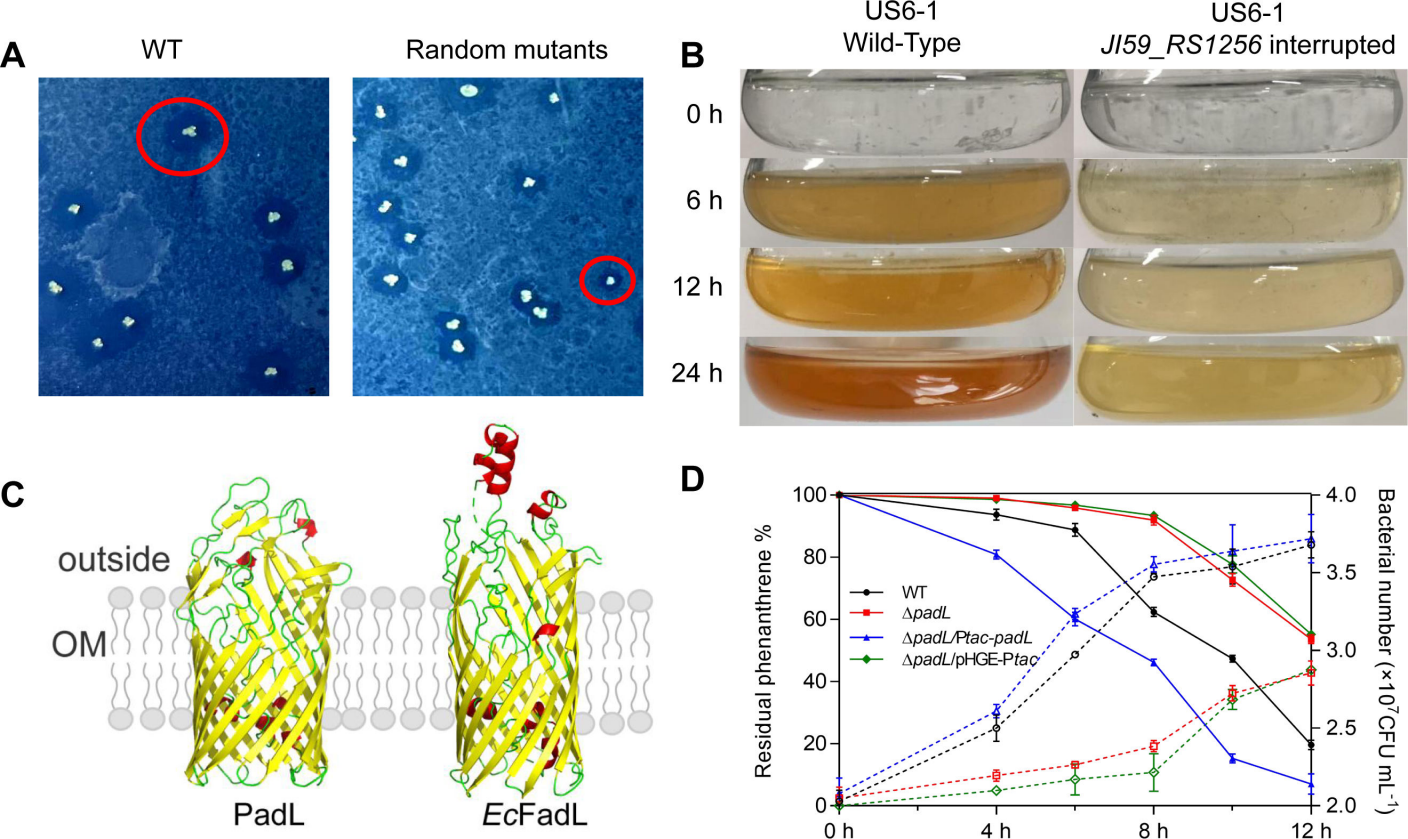
PadL (JI59_RS1256) influences the biodegradation of phenanthrene. (**A**) Screening for PAH trans-membrane transporters via transposon mutagenesis. The degradation zones of phenanthrene in the WT strain served as a control. Transposon mutants that exhibited decreased phenanthrene degradation zones compared with the WT strain were selected for the identification of transposon insertion sites. A mutant with an insertion in the JI59_RS1256 (PadL) coding region is highlighted with a red circle. (**B**) Change in culture color of the chosen transposon mutant. Cells were grown in minimal medium with 200 mg/L phenanthrene as the sole carbon source. (**C**) Protein structure of FadL from *E. coli* (PDB: 1T1L) and PadL from *N. pentaromativorans* US6-1. (**D**) Effect of PadL on phenanthrene degradation (solid lines) and growth in phenanthrene (dotted lines). Percentage of the residual phenanthrene in the minimal medium originally with 200 mg/L phenanthrene as the carbon source used by the relevant strains. Growth curves of strains in defined medium with phenanthrene as the sole carbon source. WT, wild type of *N. pentaromativorans* US6-1; Δ*padL: padL* deleted mutant; Δ*padL*/pHGE-P*tac*: Δ*padL* containing a pHGE-P*tac* empty plasmid; Δ*padL*/P*tac-padL*: complementing *padL* into Δ*padL* using *tac* promoter. The expression of the *padL* gene was driven by 0.1 mM IPTG.

*N. pentaromativorans* US6-1 has been reported to efficiently degrade phenanthrene (a representative PAH with three fused benzene rings) (24). To explore the genetic elements involved in phenanthrene biodegradation, mariner-based transposon mutagenesis (25) was performed to construct a random mutation library of strain US6-1. The resulting mutant variants were screened on minimal medium plates sprayed with phenanthrene-dissolved acetone. Strain US6-1 formed colonies surrounded by phenanthrene degradation zones (Fig. 1A). As a comparison, 86 of approximately 500 random mutant variants gained smaller phenanthrene degradation zones (Fig. 1A). The insertion sites in the mutant variants with a reduced ability to degrade phenanthrene were then identified using arbitrarily primed PCR, DNA sequencing, and BLASTn analysis. Notably, six insertions were mapped to the coding sequence of *JI59_RS12565* that encodes a FadL family protein, highlighting the involvement of JI59_RS12565 in phenanthrene degradation.

2-Hydroxybenzoate semi-aldehyde and catechol are golden-yellow intermediate metabolites of phenanthrene when degraded by strain US6-1 (26). Accordingly, the intensity of the yellow color can be used to indicate the degree of phenanthrene degradation. When the cells were grown in a minimal medium with phenanthrene as the sole carbon source, the *JI59_RS12565* interrupted mutant exhibited a lighter yellow color than the wild type of strain US6-1 (Fig. 1B). Protein structure model analysis showed that similar to *Ec*FadL, the protein JI59_RS12565 formed a 14-stranded β-barrel structure that is integrated in the outer membrane (Fig. 1C). Collectively, we named JI59_RS12565 as PadL in strain US6-1.

To further explore whether PadL is involved in phenanthrene biodegradation, the *padL* gene was in-frame deleted and the Δ*padL* strain was constructed. When cells were grown in a defined medium with phenanthrene as the sole carbon source, the Δ*padL* strain exhibited a reduced ability to degrade phenanthrene and a significant growth defect, which could be reversed by genetic complementation *in trans* (Δ*padL*/P*tac-padL*) (Fig. 1D). Notably, the deletion of the *padL* gene had no significant effect on the growth of cells when inoculated in a defined medium with sucrose as the sole carbon source (Fig. S1). These results indicate that PadL plays a role in phenanthrene biodegradation in strain US6-1.

### PadL mediates the uptake of phenanthrene

**Fig 2:**
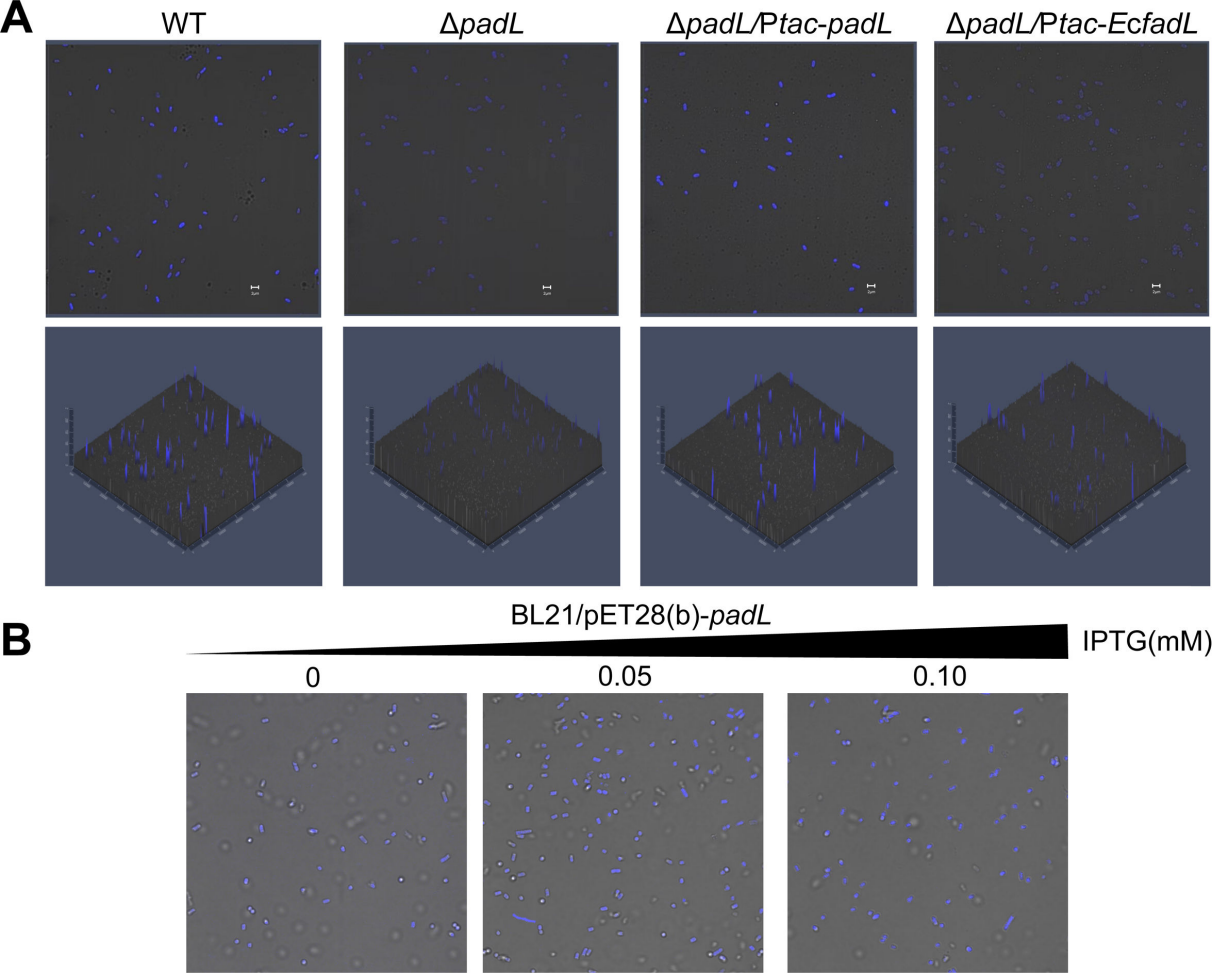
PadL plays a crucial role in phenanthrene assimilation. (**A**) Confocal microscopy images showing phenanthrene fluorescence in *N. pentaromativorans* US6-1. Upper: phenanthrene fluorescence in cells using merged filters; Lower: fluorescence intensity through 2.5D imaging. Expression of *padL* and *E. coli fadL* (*EcfadL*) is controlled by 0.1 mM IPTG. (**B**) Confocal microscopy images showing phenanthrene fluorescence in *E. coli* BL21. The expression of *padL* is controlled by varying concentrations of IPTG. WT: wild type of *N. pentaromativorans* US6-1; Δ*padL: padL* deleted mutant; Δ*padL*/P*tac-padL*: complementing *padL* into Δ*padL*; Δ*padL*/P*tac-EcfadL*: complementing *EcfadL* into Δ*padL*; BL21/pET28(b)-*padL*: expressing *padL* under the control of the T7 promoter into *E. coli* BL21. Experiments were performed at least three times, and representative results are shown.

Given that PadL shared a highly similar structure with *Ec*FadL that functions in the transport of long-chain fatty acids (22), we inferred that PadL is involved in phenanthrene biodegradation by affecting its assimilation. To determine the exact role of PadL in phenanthrene assimilation, the cells with different expression levels of *padL* were grown for 6 h in defined media with 200 mg/L phenanthrene as the sole carbon source, and laser confocal microscopy was used to observe the luminescence from intracellular phenanthrene (27). The Δ*padL* strain (the *padL*-deleted variant of strain US6-1) exhibited a diminished fluorescence relative to the wild type of strain US6-1. This phenomenon could be reversed by genetic complementation (Δ*padL*/P*tac-padL*, complementing the *padL* gene in the Δ*padL* variant). Interestingly, the expression of *EcfadL* in the Δ*padL* strain (Δ*padL*/P*tac-EcfadL*) did not restore the intracellular luminescence (Fig. 2A). All these findings suggest that PadL in *N. pentaromativorans* US6-1 may specifically mediate the uptake of phenanthrene. To prove this speculation, we further heterologously expressed the *padL* gene under the control of the *lac* operon in *E. coli* BL21 that cannot degrade phenanthrene, generating BL21/pET28b-*padL*. Next, BL21/pET28b-*padL* was incubated in LB medium supplied with 200 mg/L phenanthrene, and the expression of *padL* was induced by adding isopropyl β-D-1-thiogalactopyranoside (IPTG). Notably, IPTG induction led to the production of fluorescence signals in BL21/pET28b-*padL*. The higher the IPTG concentration was, the stronger the fluorescence signal was. However, the BL21/pET28b-*padL* cells without IPTG induction also yielded weak fluorescence signals, probably due to a leaky expression of the recombinant plasmid (Fig. 2B). Therefore, *E. coli* BL21 can be endowed with an ability to assimilate phenanthrene after heterologous expression of PadL. Collectively, these results indicate that PadL indeed mediates the uptake of phenanthrene.

### PadL affects the expression of the phenanthrene-degrading gene

**Fig 3:**
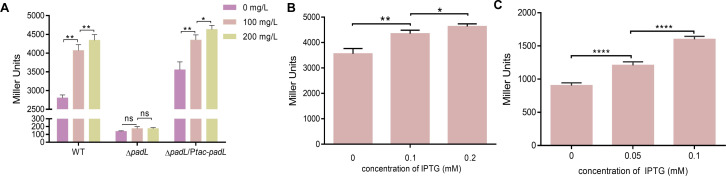
Effect of PadL on the expression of *ahdA1e*. (**A**) A *lacZ* reporter system was used to determine the activity of the *ahdA1e* promoter in the strains of *N. pentaromativorans* US6-1 cultured in P5Y3 medium with 0, 100, or 200 mg/L phenanthrene. (**B**) A *lacZ* reporter system was used to determine the activity of the *ahdA1e* promoter in *N. pentaromativorans* US6-1 cultured in P5Y3 medium with 200 mg/L phenanthrene. *N. pentaromativorans* US6-1 expressed *padL* under varying concentrations of IPTG. (**C**) A *lacZ* reporter system was used to determine the activity of the activity of the *ahdA1e* promoter in *E. coli* BL21 cultured in LB medium with 200 mg/L phenanthrene. *E. coli* BL21 expressed *padL* under varying concentrations of IPTG. WT, wild type of *N. pentaromativorans* US6-1; Δ*padL, padL* deleted mutant; Δ*padL*/P*tac-padL,* complementing *padL* into Δ*padL*. Asterisks indicate statistical significance: *, *P* < 0.05; **, *P* < 0.01; ***, *P* < 0.001; ****, *P* < 0.0001; ns, not significant.

Our previous studies have shown that phenanthrene availability affects the expression level of *ahdA1e*, a gene encoding the *α* subunit of an aromatic ring-hydroxylating dioxygenase, a pivotal enzyme for PAH degradation in *N. pentaromativorans* US6-1 (26). We wondered whether PadL is responsive to the expression of *ahdA1e*. For this purpose, the promoter of the *ahdA1e* gene was integrated into a *lacZ*-reporter system, and the activity of the promoter was evaluated based on the β-galactosidase activity (28). The results in Fig. 3A showed that the activity of the *ahdA1e* promoter is, indeed, positively correlated with the dose of phenanthrene in the wild-type strain. However, the activity of the *ahdA1e* promoter cannot be induced by phenanthrene in the Δ*padL* strain. This phenomenon can be reversed by genetic complementation (Δ*padL*/P*tac-padL*). These findings suggest that the deletion of *padL* leads to a reduced availability of intracellular phenanthrene, thereby diminishing the expression of PAH-degrading genes.

To further demonstrate the function of PadL in the *ahdA1e* expression, the activity of the *ahdA1e* promoter in Δ*padL*/P*tac-padL* under the control of the *tac* promoter (Fig. 3B) and BL21/pET28b-*padL* under the control of the T7 promoter (Fig. 3C) was examined. These cells were exposed to varying IPTG concentrations for inducing the *padL* expression in P5Y3 or LB medium containing 200 mg/L phenanthrene. Transcription levels of *ahdA1e* increased with the presence of IPTG, verifying that the changes in transcription levels resulted from PadL expression. Besides, we also confirmed that expressing the *ahdA1e* gene in the Δ*padL* variant cannot compensate for the degradation of phenanthrene (Fig. S2). Collectively, we conclude that PadL facilitates the assimilation of phenanthrene.

### PadL may specifically transport PAHs

**Fig 4:**
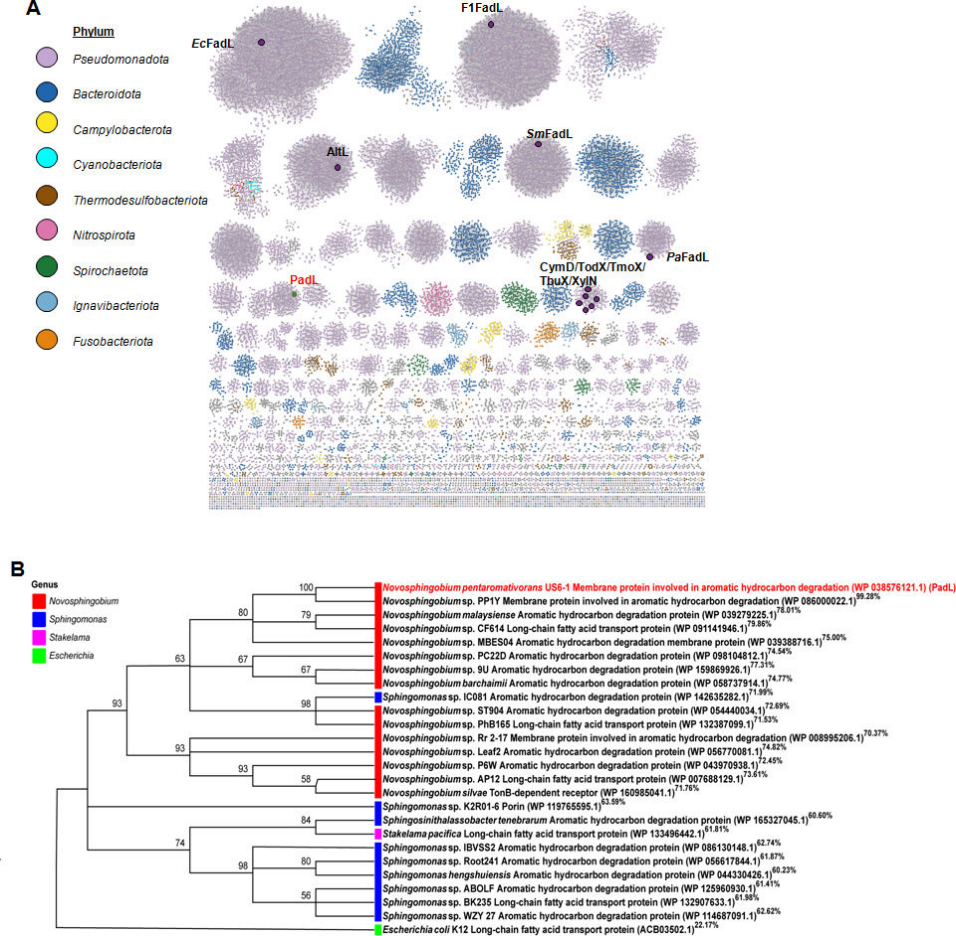
PadL is a novel subfamily of the FadL family predominantly distributed among PAH-degrading bacteria. (**A**) A sequence similarity network (SSN) of the FadL family. The SSN was constructed using EFI-EST with 26,762 sequences from OM transport protein (OMPP1/FadL/TodX) (IPR005017) (Supplementary excel file 1) and analyzed with Cytoscape. The generated SSN reveals multiple clusters with alignment scores ranging from 111 to 334 (sequence identity >47%). Each node represents a protein and is colored based on its classification. PadL is labeled in red, and other proteins reported are labeled in black. (**B**) Phylogenetic tree analysis of PadL. The upper right corner is marked with the results of sequence homology alignment of PadL with BLASTP (Identity %).

Multiple sequence alignment indicated that PadL has low homology with the known FadL homologs capable of transporting fatty acids and other hydrophobic hydrocarbon compounds (Table 1). To explore the relationship between PadL proteins, we applied the Enzyme Function Initiative (EFI) to analyze the distribution and sequence similarity networks (SSNs) of FadL family proteins. Consistent with low sequence similarities among FadL family proteins, *Np*FadL homologs are organized into a large number of clusters (Fig. 4A). Interestingly, PadL is distinguishable from documented FadL family proteins, which are distributed across six distinct clusters (Fig. 4A), implying that PadL family proteins have evolved to cope with diverse and distinct substrates despite their overall conserved structure. Analyses of their evolutionary relationship revealed that PadL is distantly related to TodX, a representative of the monoaromatic hydrocarbons (MAHs) transport proteins, and to *Ec*FadL, a representative of the LCFA transport proteins, suggesting an early divergence in their function (Fig. S3). Importantly, PadL showed a high similarity with FadL homologs found in PAH-degrading bacteria, such as the *Sphingomonas* and *Novosphingobium* genera (Fig. 4B). Collectively, PadL represents a novel subfamily of FadL family channel proteins predominantly distributed among PAH-degrading bacteria.

**TABLE 1:** Comparison of PadL with other FadL family channel proteins

Protein	Organism	Substrate(s)	Length*^a^*	Identity^*b*^
TodX	*P. putida* F1	MAHs^*c*^	453	<15%
TmoX	*P. oleovorans*	MAHs	480	24.85%
AltL	*A. venetianus* RAG-1	Long-chain alkanes	554	21.46%
*Ec*FadL	*E. coli*	Long-chain fatty acids	446	22.17%

^*a*^ aa, amino acids.

^*b*^ Identities were calculated using BLASTP.

^*c*^ MAHs include monoaromatic hydrocarbons benzene, toluene, ethylbenzene, and xylene.

**Fig 5:**
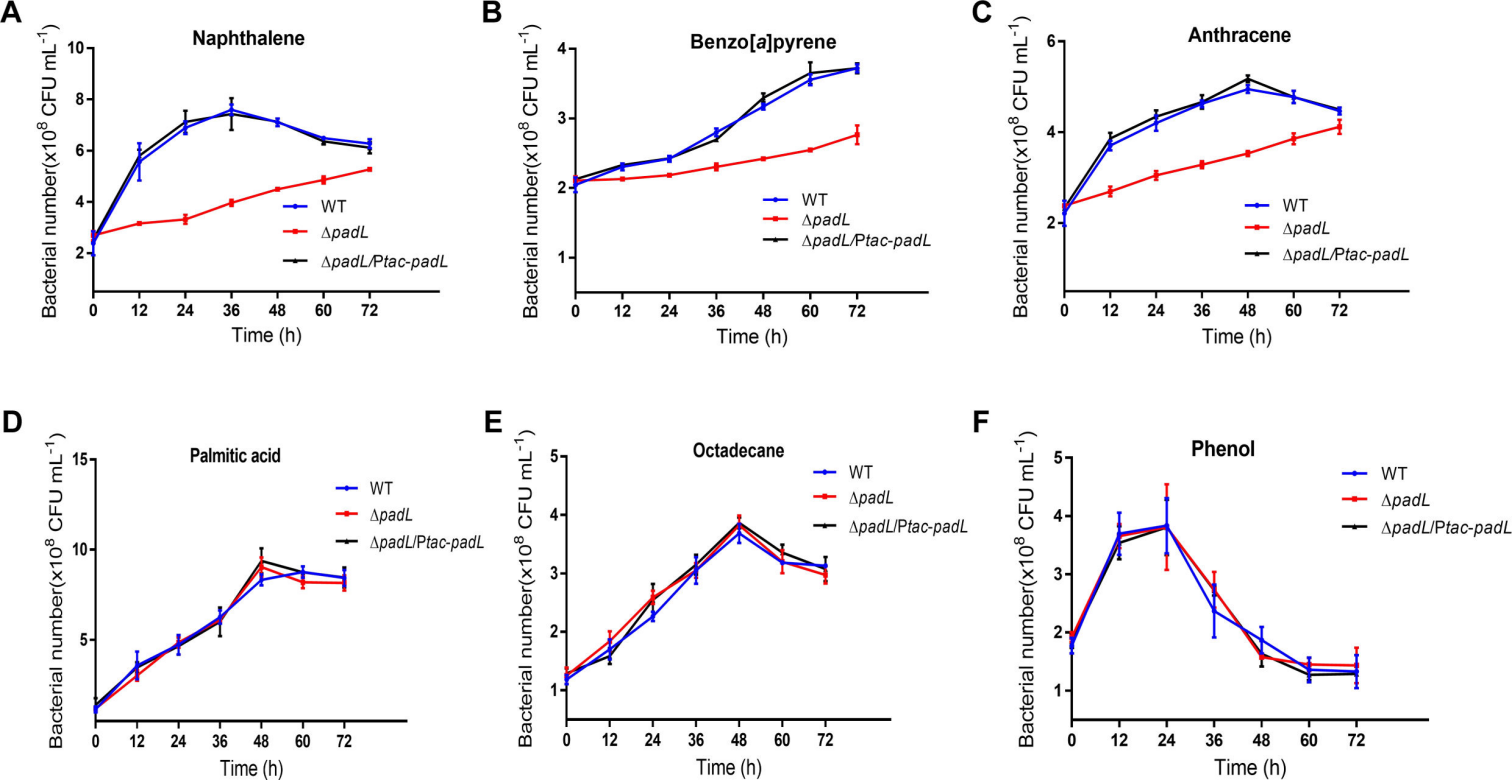
PadL specifically transports PAHs. Growth curves of strains cultured in the defined medium with naphthalene (**A**), benzo[*a*]pyrene (**B**), anthracene (**C**), palmitic acid (**D**), octadecane (**E**), or phenol (**F**) as the sole carbon source. WT, wild type of *N. pentaromativorans* US6-1; Δ*padL, padL* deleted mutant; Δ*padL*/P*tac-padL,* complementing *padL* into Δ*padL*. The expression of *padL* is controlled by the addition of 0.1 mM IPTG.

The substrate specificity of PadL was determined by measuring the cell growth of the wild type, Δ*padL*, and Δ*padL*/P*tac-padL* variants of strain US6-1 in defined media with PAHs and other potential FadL family transporter substrates as the sole carbon source, such as LCFA (e.g., palmitic acid), long-chain alkanes (e.g., octadecane), and other aromatic compounds (e.g., phenol). The results showed that the deletion of the *padL* gene significantly impaired the growth of cells when cultured in a defined medium with naphthalene, benzo[*a*]pyrene, or anthracene as the sole carbon source. This phenomenon can be reversed by genetic complementation of the *padL* gene *in trans* (Fig. 5A through C). Conversely, the Δ*padL* variant did not exhibit growth impairment in a defined medium with possible substrates of FadL family transporters, including long-chain fatty acids, long-chain alkanes, or monocyclic aromatic hydrocarbons (such as palmitic acid, octadecane, or phenol) as the sole carbon source (Fig. 5D through F). Therefore, we conclude that the deletion of the *padL* gene impairs the transport of PAHs but not non-aromatic compounds.

### Substrate-binding pocket of PadL affects PAH transport

**Fig 6:**
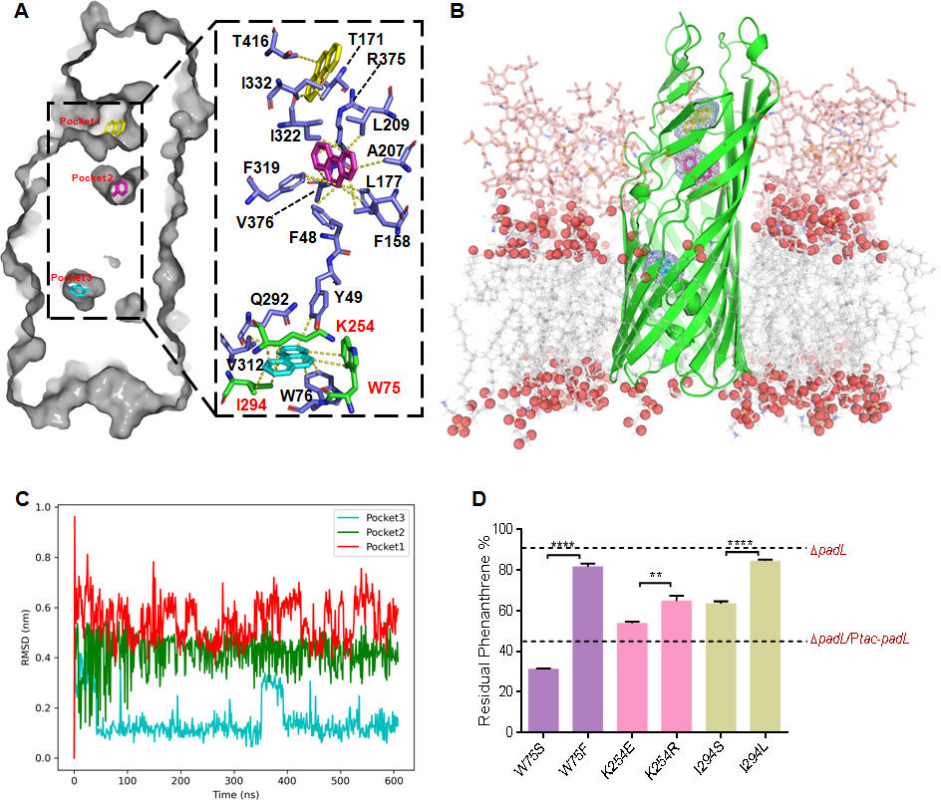
Substrate-binding pocket of PadL affects the efficiency of phenanthrene transport. (**A**) Molecular docking between substrate phenanthrene and PadL. The residues interacting with ligands are shown as sticks and are labeled. The residues that have been experimentally proven to be effective are highlighted in different colors and labeled with red text. Phenanthrene is colored yellow, violet, and blue. (**B**) The initial view of PadL embedded in the OM with LPS, consisting of hydrophobic lipids A and the hydrophilic cores in the outer leaflet and phospholipids in the inner leaflet. Within the LPS structure, hydrophilic cores are depicted as transparent pink sticks, with hydrogen bonds hidden to enhance clarity. Lipid A of LPS and phospholipids are illustrated as gray sticks, with oxygen atoms represented as red spheres. (**C**) The RMSD (root mean square deviation) values of the ligands in three pockets are represented in different colors. (**D**) Percentage of the residual phenanthrene in the minimal medium originally with 200 mg/L phenanthrene as the sole carbon source used by the relevant Δ*padL* strain and site-directed PadL-mutated variants for 24 h. The percentage of residual phenanthrene used by the Δ*padL* and Δ*padL*/P*tac-padL* strains used as a control. Δ*padL, padL* deleted mutant; Δ*padL*/P*tac-padL,* complementing *padL* into Δ*padL*. The expression of *padL* and its variants is controlled by the addition of 0.1 mM IPTG. Asterisks indicate statistical significance: **, *P* < 0.01; ***, *P* < 0.001; ****, *P* < 0.0001.

To elucidate the molecular mechanism of PadL in transporting PAHs, a molecular docking program was performed to study the interactions between PadL and phenanthrene. The results in Fig. 6A showed that phenanthrene was well fitted with three hypothesized substrate-binding pockets and that the distribution of these pockets could align with the classical pathway model (22). Similar to phenanthrene, benzo[*a*]pyrene also had a potential interaction with the hydrophobic substrate-binding pocket of PadL (Fig. S4). Subsequently, we developed a model based on the docking results (Fig. 6B) by incorporating the protein, phenanthrene, OM, and LPS, followed by molecular dynamics (MD) simulations. The results (Fig. 6C) indicated that pocket 2 and pocket 3 constituted relatively stable environments for phenanthrene. The potential pathway between pocket 2 and pocket 3 of PadL for phenanthrene transport was also identified (Movie S1).

We also calculated the binding free energy of phenanthrene in pocket 3 using gmx_MMPBSA. The simulated binding energy between phenanthrene and PadL was around −12 kcal/mol, and residues Trp75, Lys254, and Ile294 seemed to contribute mostly to the binding (Fig. S5). These three key residues (W75, K254, and I294) were subsequently chosen for site-directed mutagenesis. Given the acid-base properties and spatial configuration, we mutated these three substrate-binding pocket residues to other amino acids with similar (W75F, K254R, and I294L) or different (W75S, K254E, and I294S) properties. The mutated *padL* genes were expressed in the Δ*padL* variant using the pHGE-P*tac* plasmid. The resulting variants were then grown for 24 h in defined media containing 200 mg/L phenanthrene as the sole carbon source. Residual phenanthrene levels were quantified. While most of these PadL variants exhibited significantly higher residual phenanthrene, the PadL^W75S^ variant gave lower residual phenanthrene than the control expressing the native *padL* gene (Fig. 6D). This may be explained by that the substantial size reduction by the residue replacement may enlarge the pocket and passage for phenanthrene. Collectively, these results demonstrate that the hydrophobic amino acid residues within substrate-binding pocket 3 of PadL play a crucial role in phenanthrene transport.

## DISCUSSION

Despite the bacterial remediation of PAHs having attracted considerable attention in recent decades, most of the studies have focused on the processes before and after trans-membrane transport. Trans-membrane transport of PAHs is a pivotal intermediary phase that connects PAH adsorption and degradation inside the cell, but little research has been done in this area. There is still a pronounced gap in our understanding of the transport proteins that facilitate the traffic of PAHs across membranes.

In this study, a FadL family transporter was identified as a potential candidate largely responsible for the assimilation of PAHs in *N. pentaromativorans* US6-1. We found that PadL deficiency substantially impaired the transport and degradation of phenanthrene in *N. pentaromativorans* US6-1 (Fig. 1 and 2). In addition to PadL, TBDT-11 has been shown to play a contributory role in the trans-membrane transport of PAHs as the absence of the TBDT-11 transporter significantly reduced the intracellular fluorescence intensity of both benzo[*a*]pyrene and phenanthrene in *N. pentaromativorans* US6-1 (27). Further investigation is required to elucidate whether PadL and TBDT exhibit compensatory or overlapping functions in the trans-membrane transport of PAHs.

The FadL family is essential in the cross-membrane transport of a variety of hydrophobic molecules, including LCFAs (22, 29), MAHs (8), and medium-chain and long-chain alkanes (23, 30). Many bacteria contain multiple FadL-type proteins. For example, there are three in *Pseudomonas aeruginosa* and *Vibrio cholerae*. Somboon et al. investigated the transport capabilities of three FadL family transporters (TodX, CymD, and *Pp*FadL) in *P. putida* PpF1 and demonstrated that TodX and CymD can transport MAHs. In contrast, *Pp*FadL lacks this capability and is exclusively restricted to the translocation of LCFAs (20). Based on these findings, it has been proposed that the FadL family transporters have a pronounced substrate specificity (20). In this study, we found that the FadL homologs are widely spread and organized into a large number of clusters based on evolutionary analyses. This can be readily explained by rather low sequence similarity. In contrast, most, if not all, of FadL homologs adopt a similar β-barrel structure. While these characteristics substantiate that all FadL homologs have a transporter activity, only those sharing a considerable high-level sequence similarity may transport the same substrate. In line with this, *Np*PadL could be a novel subfamily of PadLs, and this transporter group can be found in only certain genera of PAH-degrading bacterial, including *Sphingomonas* and *Novosphingobium* (Fig. 4).

PadL has three predicted substrate binding pockets to interact with phenanthrene (Fig. 6A). Although we have demonstrated an important role of pocket three in phenanthrene cross-membrane transport, it remains uncertain whether all three pockets can function under different conditions. We hypothesized that the activation status of these pocket channels may be influenced by the environmental conditions in which the strains exist.

**Fig 7:**
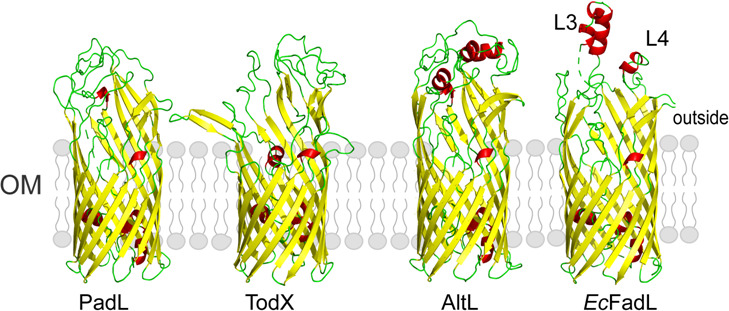
Structural comparison of FadL family members. Extracellular loops are colored red (**L3 and L4**).

The crystal structure analysis reveals that *Ec*FadL (PDB ID: 1T1L) is composed of a 14-stranded β-barrel. The N-terminus of this protein forms a small and compact structural domain, featuring three brief helices that effectively occlude the interior of the β-barrel. Between the two extracellular loops, L3 and L4, there lies an exposed hydrophobic loop that provides the initial interaction site with a high affinity for free hydrophobic compounds (20, 22) (Fig. 7). This site is posited to capture environmental hydrophobic molecules, thereby facilitating the formation of a locally high substrate concentration. A comparative analysis of the PadL protein with homologs from *Ec*FadL, as well as other documented FadL family members, indicates that although the core β-barrel structure is conserved, the L3 and L4 loops are missing in PadL (Fig. 7). It is speculated that this structural variation could be associated with difference in substrate specificity.

In summary, our findings offer valuable insights into the molecular mechanisms underlying the trans-membrane transport of PAHs by the FadL transporter. This could have significant implications for PadL in PAH bioremediation and expand our understanding of how organisms metabolize complex organic pollutants.

## MATERIALS AND METHODS

### Strains, plasmids, media, and culture conditions

The strains and plasmids used in this study are listed in Table S1. *E. coli* cells were grown in Luria-Bertani (LB) medium (5 g/L yeast extract, 10 g/L tryptone, and 10 g/L NaCl; 2% agar added for solid medium) at 37°C. The *E. coli* WM3064 culture (LB) was supplemented with 0.3 mM diaminopimelic acid (DAP) .

*N. pentaromativorans* US6-1 was grown in P5Y3 medium (25 g/L marine salt, 5 g/L bacteriological peptone, and 3 g/L yeast extract; 2% agar added for solid medium) at 30°C (31). For growth measurement, *N. pentaromativorans* strains were cultured in marine minimal medium with various carbon sources (32). For the PAH biodegradation assay, *N. pentaromativorans* US6-1 was grown in marine minimal medium with phenanthrene (200 mg/L) as the sole carbon source at 30°C as described previously (26).

When antibiotics were needed in the medium, the final concentrations were set at 50 µg/mL for both gentamicin (Gm) and kanamycin (Km), and 5 mg/mL for streptomycin (Str). When necessary, varying concentrations of IPTG were added to induce gene expression.

### Transposon insertion mutagenesis

As previously described, we used plasmid pHGT02, a derivative of the broadly applied mariner-based transposon vector pFAC, to construct a library of random mutations (25). The plasmid pHGT02 was conjugatively transferred from *E. coli* WM3064 to *N. pentaromativorans* US6-1. Subsequently, the transconjugants were spread onto P5Y3 agar plates supplemented with gentamicin. Once colonies became discernible, 5 mg/mL phenanthrene solution was sprayed onto the plates, which were then incubated at 30°C. Colonies that exhibited a smaller hydrolysis circle were selected for further studies (33). The arbitrarily primed PCR method was used to identify the location of transposon insertions in the selected colonies as described previously (34).

### Construction of gene deletion mutants

For *N. pentaromativorans* US6-1, in-frame gene deletion strains were created with an *rpsL*-based tag-free gene deletion system, as described previously (28, 32). Briefly, two fragment flanking genes of interest (500–800 bp) were amplified using PCR with primers (5O/5I primers and 3I/3O primers, Table S2) carrying the restriction enzyme sites. Then, the two fragments were fused in a second round of PCR with 5O/3O primers and ligated into the pAK405 plasmid. The recombinant plasmid was transformed into *E. coli* WM3064 (DAP auxotroph) and subsequently transferred into *N. pentaromativorans* US6-1 through conjugation. PCR validation was conducted using LF/SR and SF/LR primers (Table S2) to confirm the integration of the pAK405 recombinant plasmid into the chromosome of *N. pentaromativorans* US6-1. After obtaining the resulting colonies, they were restreaked on P5Y3 agar plates containing 5 mg/mL of streptomycin, or 50 µg/mL of kanamycin, if needed. The kanamycin-sensitive colonies were screened for the deletion of the target gene using PCR with LF/LR primers. Finally, the deletion mutants were verified by DNA sequencing.

### Genetic complementation and gene overexpression

The IPTG-inducible plasmid pHGE-P*tac* with broad-host range was used for genetic complementation and gene expression/overexpression of *N. pentaromativorans* strains (35). For *E. coli* strains, another IPTG-inducible plasmid (pET-28b) was used for the expression of *padL*. Briefly, the target fragment was amplified using PCR and cloned into the pHGE-P*tac* or pET-28b plasmid. The recombinant plasmid was maintained in *E. coli* WM3064 and then transferred into the relevant *N. pentaromativorans* and *E. coli* strains via conjugation. To generate the W39S/W39F/K218E/K218R/I258S/I258L mutation for *N. pentaromativorans* PadL, pHGE-P*tac-padL* was used as the template for site-directed mutagenesis according to the previously described method (36). Mutated *padL* was amplified using PCR, digested with restriction enzymes, and then transformed into *E. coli* WM3064. The resulting plasmids were transferred into the *padL* deleted strain via conjugation. Sequencing was performed to confirm the successful construction of the complementation or overexpression strain.

### Measurement of residual phenanthrene

The residual phenanthrene in the medium was analyzed using high-performance liquid chromatography (HPLC) as described previously (28, 37). First, the sample was extracted with ethyl acetate, and the extract was evaporated. Then, the extracts were dissolved in methanol, filtered, and analyzed by HPLC. For measurement, an InertsilODS-3 C18 column (4.6 × 250 mm, 5 µm) was used, and the mobile phase consisted of methanol and water (80:20, vol:vol) set at a flow rate of 1.0 mL/min, using the Waters 2996 dual-wavelength detector at 254 nm.

### Observation via laser confocal microscopy

Bacterial uptake of phenanthrene and benzo[*a*]pyrene was observed using a laser scanning confocal microscopy (27). *N. pentaromativorans* and *E. coli* strains were grown in P5Y3 medium and LB medium, respectively, to stationary phase and then washed three times with minimal medium. The cells were transferred to the appropriate fresh medium with 200 mg/L phenanthrene or benzo[*a*]pyrene. The strains were incubated to an OD_600_ of 0.4 at 30°C or 37°C. Then, a laser scanning confocal microscope was used to observe the fluorescence intensity of cultures. Phenanthrene and benzo[*a*]pyrene were excited with a 405 nm laser, and the emission was set at 450–550 nm (38).

### Measurement of cell growth

For growth assessment, the marine minimal medium supplied with different carbon sources was used in this study. Fresh medium was inoculated with overnight P5Y3 cultures grown from a single colony at a 1:100 dilution. Samples were collected every 12 h, and bacterial liquid was subjected to 10-fold dilution before spreading 100 µL of the suspension on plates, with three replicates per sample. After incubation at 30°C for 48 h, colony-forming units were counted.

### Expression assay

The expression of genes of interest was evaluated using an integrated *lacZ*-reporter system (39). Fragments of approximately 500 bp containing the promoter sequences were cloned into the reporter vector pHGER01 to generate transcriptional fusions based on promoter prediction. These vectors were verified by sequencing before being transferred into appropriate strains by conjugation. Mid-exponential phase cultures were harvested, appropriately aliquoted, and subjected to a β-galactosidase activity assay, as previously described (28).

### Bioinformatics and molecular docking

The EFI-Enzyme Similarity Tool (EFI-EST) (https://efi.igb.illinois.edu) was used to generate Sequence similarity networks (SSNs) for representative sequences of the FadL family (40, 41). 26,762 sequences were input into the EFI-EST to generate the network analysis; Cytoscape 3.9.1 was used to generate figures. All networks used for analysis and in the figures were 100% representative node networks. Furthermore, the MUSCLE alignment method and MEGA11 were used to construct an unrooted neighbor-joining phylogenetic tree of proteins in the SSN. The evolutionary history was represented by the bootstrap consensus tree inferred from 1,000 replicates. Numbers at nodes represent the bootstrap values.

AutoDock Vina was used to conduct molecular docking, and Pymol was used to generate protein structure images. PLIP was used to predict interactions between ligands and residues (42). A BLASTp search was performed in the NCBI database (43). The protein model of PadL was obtained from the AlphaFold DB (G6EAN6). Based on structural comparisons with PadL homologs proteins in the PDB, sequence annotations from InterPro (44), and the confidence level of the AlphaFold2 (AF2)-predicted structure (because the signal peptide lacks co-evolution with residues, it scores lower) (45), we excised the signal peptide (residues 1–45) from the AF2-predicted structure.

### Model building

Because FadL in *P. aeruginosa* (*Pa*FadL) is the most structurally similar homologous protein to PadL, and given that *P. aeruginosa* has a well-developed OM model (46), we integrated the AF2-predicted PadL, lacking a signal peptide, along with three phenanthrenes, into the *P. aeruginosa* OM, using LPS composition on the outer leaflet. We followed the detailed procedural guide provided in “Membrane Builder” via CHARMM-GUI to incorporate the protein into asymmetric OM structures (47, 48). Moreover, a planar region representing a 150 mM KCl solution, with a thickness of 2.25 nm along the *Z*-axis at the top and bottom of a rectangular box, was used to mimic the physiological bulk ion concentration.

### MD simulation

MD simulation was conducted using GROMACS-v2023 (49, 50), with the all-atom CHARMM36m force field for lipids, ions, protein, LPS, and TIP3P water. The “Ligand Reader & Modeler” tool within CHARMM-GUI was used to generate the CGenFF topology for phenanthrene. Initial systems underwent energy minimization over the 3,000 steepest descent steps and gradual equilibration in the NPT ensemble at 310 K, following the equilibration protocol provided by CHARMM-GUI. Following equilibration simulations, a 600 ns production run was conducted in the NPT ensemble at 310 K and 1 bar pressure. Temperature and pressure were coupled using the velocity-rescale method (time constant of 1 ps) and semi-isotropic pressure coupling with the Parrinello-Rahman algorithm (time constant of 5 ps), respectively.

### Statistical analysis

Unless otherwise stated, all the data presented are the results from three independently repeated experiments. Student’s *t*-test was performed for pairwise comparison. Values were presented as means and standard errors of the mean (SEM).

## SUPPLEMENTAL MATERIAL

Supplemental material aem.00827-24-s0001.pdf Tables S1 and S2; Fig. S1 to S5.

Supplemental Excel file aem.00827-24-s0002.xlsx Sequence alignment.

Movie S1 aem.00827-24-s0003.mp4 Potential pathway between pocket 2 and pocket 3 of PadL for phenanthrene transport.

ASM does not own the copyrights to Supplemental Material that may be linked to, or accessed through, an article. The authors have granted ASM a non-exclusive, world-wide license to publish the Supplemental Material files. Please contact the corresponding author directly for reuse.
