# Supplementary material for: A novel FadL family outer membrane transporter is involved in the uptake of polycyclic aromatic hydrocarbons
Source: Appl Environ Microbiol. 2025 Jan 24;91(2):e00827-24. doi: 10.1128/aem.00827-24 (PMC11837497; doi:10.1128/aem.00827-24)
Supplement: Supplemental material — Tables S1 and S2; Fig. S1 to S5. [file aem.00827-24-s0001.pdf]

Supplementary Materials

A novel FadL family outer membrane transporter is involved in the uptake of polycyclic aromatic hydrocarbons

Qiu Meng[a], Yuxuan Liang[b], Yinming Xu[a], Saiyue Li[a], Haiyan Huang[a], Yuanyou Xu[b], Feifei Cao[c], Jianhua Yin[a], Tingheng Zhu[a], Haichun Gao[b]*, Zhiliang Yu[a]*

[a]College of Biotechnology and Bioengineering, Zhejiang University of Technology, Hangzhou 310014, Zhejiang Province, China

[b]Institute of Microbiology, College of Life Sciences, Zhejiang University, Hangzhou, 310012, Zhejiang Province, China

[c]Hangzhou Chuhuan Science and Technology Co., Ltd., Hangzhou 310015, Zhejiang Province, China

*__Corresponding authors__:

Haichun Gao and Zhiliang Yu

Email: haichung@zju.edu.cn (HG); zlyu@zjut.edu.cn (ZY)

Running title: PadL mediates PAH uptake

Keywords: polycyclic aromatic hydrocarbons, FadL family, trans-membrane transport, biodegradation, *Novosphingobium pentaromativorans*

Table S1 Strains and plasmids used in this study

Strain and plasmid	Description	Source or reference
Escherichia coli strains		
DH5α	Host strain for plasmids	Lab stock
WM3064	Donor strain for conjugation	TransGen Biotech
BL21	Strain for gene expression	Lab stock
BL21/pET28(b)-*padL*	Expression of *padL* gene in BL21	This study
N. pentaromativorans strains		
US6-1	Wild-type	Lab stock
Δ*padL*	Mutant of strain with deleted *padL*	This study
Δ*padL*/P*tac-padL*	Expression of *padL* in Δ*padL*	This study
Δ*padL*/P*tac-EcfadL*	Expression of *EcfadL* gene in Δ*padL*	This study
Plasmids		
pAK405	Km^r, suicide vector for sphingomonad	Kaczmarczyk et al. (2012)
pHGT02	Gm^r, mariner-based transposon vector, pFAC removing the Ap^r gene	Lou et al. (2022)
pHGE-P*tac*	Km^r, IPTG-inducible P*tac* expression vector	Meng et al. (2018)
pHGEI03	Km^r, *lacZ* reporter vector	This study
pHGEI03-P_{ahdA1e}	For measuring *ahdA1e* promoter activity	This study
pHGEI03-P_{xylE}	For measuring *xylE* promoter activity	This study

Table S2 Primers used in this study

Primer	Restriction enzyme	Nucleotide sequence (5’ to 3’)
padL deletion		
padL-LF		CAGCCGACAATCAGGTCCTC
padL-LR		AGACGTTGTAACCCCCACAC
padL-5O	*Bam*H I	AATCCGGACCGTTCGAAACA
padL-5I		AGTAGTTCAGCACCGTCGTC
padL-3O	*Hin*d III	AAATCGAAGGCACGTTCGATACCGCGCTGGAAAGGAA
padL-3I		GAACGTGCCTTCGATTTCGCGGGTGAAGTTCTGATGA
padL-SF		TACAAGGCCACGATCACACC
padL-SR		AAATCTATGGCGGGCCACTC
padL complementation and overexpression		
P*tac*-*padL*-F	*Eco*R I	ATGAAGTTGACGTCCGGC
P*tac*-*padL*-R	*Hin*d III	TCTCCGCGGCGCTCATCA
pET28b-*padL*-F		AGGATCCAAGCTTGGCTGTTT
pET28b-*padL*-F		CGAGCTCCATGAATTCTCTCCT
EcfadL expression		
P*tac*-$_{Ec}$*fadL*-F		atcgCAGGCATTCTACCTTCAGGAACA
P*tac*-$_{Ec}$*fadL*-R		aacagccaagcttggatcctTCAGAACTTCACCCGCGCG
Promoter activity		
P_{ahdA1e}-F	*Bam*H I	TGTTGGTGGCTGTAGTGAGTTG
P_{ahdA1e}-R	*Hin*d III	gtaatcatggTCATGGCTCTCTCCGGTTTCACG
P_{xylE}-F	*Bam*H I	GACCTTGTCCAGTCGCGTTA
P_{xylE}-R	*Hin*d III	gtaatcatggTCATTTCAGTTCTCCTTGTCCCTTCA
site-directed mutation		
W39S-F	*Bam*H I	CGTCGATTCGCTGTCCTGGAA
W39S-R	*Hin*d III	CCGGGTTCCAGGACAGCG
W39F-F	*Bam*H I	GGCGTCGATTCGCTGTTTTGGA
W39F-R	*Hin*d III	GGCCGGGTTCCAAAACAGC
K218E-F	*Bam*H I	TGGCGTCTCCTACGAATCGAG
K218E-R	*Hin*d III	TGCTTGATGCTCGATTCGTAGG
K218R-F	*Bam*H I	GCTTGGCGTCTCCTACAGATCGA
K218R-R	*Hin*d III	GCTTGTGCTTGATGCTCGATCTGTAG
I258S-F	*Bam*H I	TGGCAGGCATCCGGCAG
I258S-R	*Hin*d III	CGGACGCTGCCGGATGC
I258L-F	*Bam*H I	CCGTGGCAGGCATTGGGCA
I258L-R	*Hin*d III	CGGACGCTGCCGTTTGCC

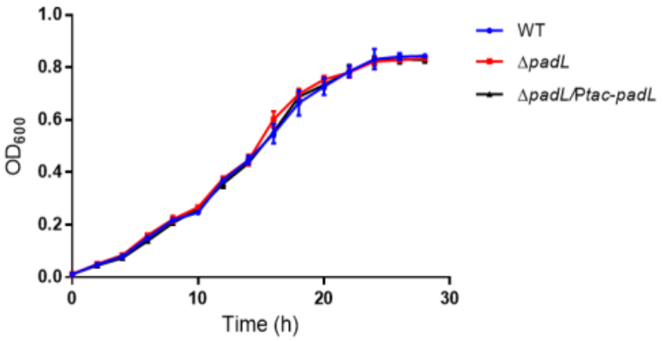

Figure S1 Growth curves of strains grown in defined media with sucrose as the sole carbon source. WT: wild type of *N. pentaromativorans* US6-1; Δ*padL*: *padL* deleted mutant; Δ*padL*/P*tac-padL*: complementing *padL* into Δ*padL*. Expression of the *padL* gene is controlled by the addition of 0.1 mM IPTG.

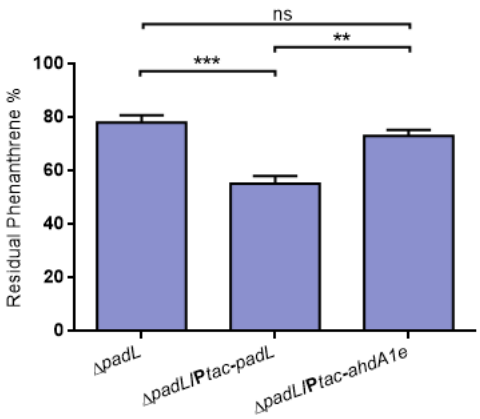

Figure S2 Residual phenanthrene from defined media with 200 mg/L phenanthrene as the sole carbon source for cell growth. Δ*padL*: *padL* deleted mutant; Δ*padL*/P*tac-padL*: complementing *padL* into Δ*padL*; Δ*padL*/P*tac-ahdA1e*: complementing *ahdA1e* into Δ*padL*. Expression of the *padL* and *ahdA1e* genes is controlled by the addition of 0.1 mM IPTG. The residual phenanthrene was measured after degradation for 8 hours.

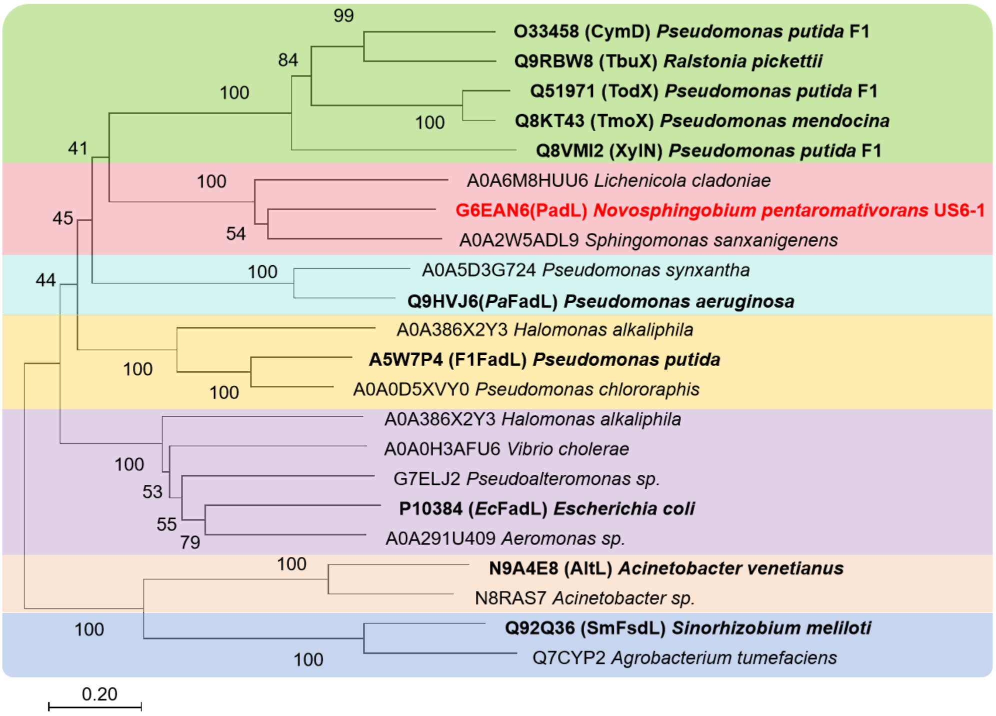

Figure S3 Phylogenetic tree analysis of PadL. An unrooted Neighbor-joining phylogenetic tree of proteins in the SSN was constructed using the MUSCLE alignment method and MEGA11. The evolutionary history was represented by the bootstrap consensus tree inferred from 1000 replicates. Numbers at nodes represent the bootstrap values.

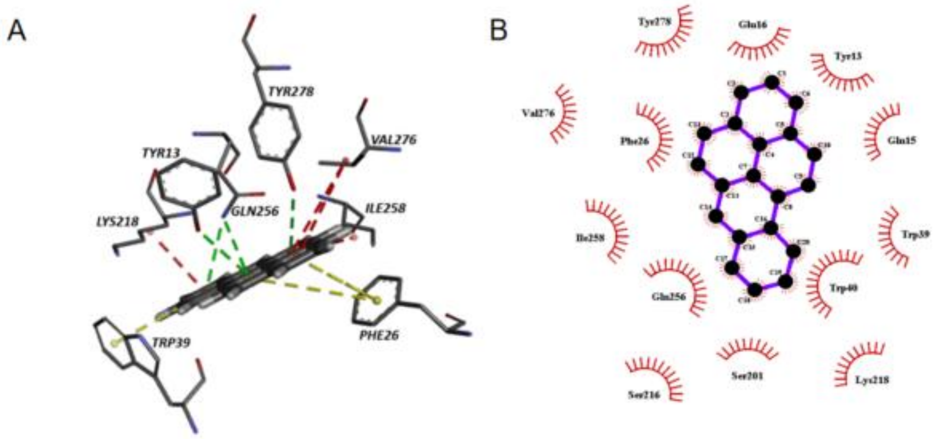

Figure S4 Molecular docking between substrate benzoa[*a*]pyrene and PadL. (A) Benzoa[*a*]pyrene is combined with PadL. (B) Benzoa[*a*]pyrene (purple) engages in hydrophobic interactions.

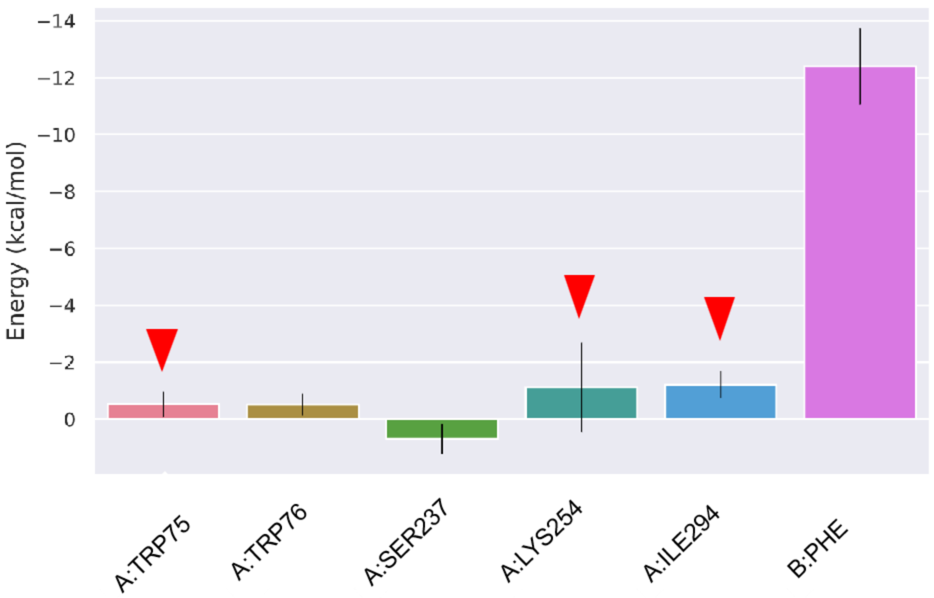

Figure S5 Binding free energy of phenanthrene in pocket 3 computed by using gmx_MMPBSA. The residues that have been experimentally proven to be effective are marked by red arrows. A: receptor protein (PadL); B: ligand (phenanthrene, PHE).

References

Kaczmarczyk A, Vorholt JA, Francez-Charlot A. 2012. Markerless gene deletion system for Sphingomonads. Appl Environ Microbiol 78:3774–3777.

Lou J, Cai J, Hu X, Liang Y, Sun Y, Zhu Y, Meng Q, Zhu T, Gao H, Yu Z, Yin J. 2022. The stringent starvation protein SspA modulates peptidoglycan synthesis by regulating the expression of peptidoglycan synthases. Mol Microbiol 118(6):716–730.

Meng Q, Sun Y, Gao H. 2018. Cytochromes *c* constitute a layer of protection against nitric oxide but not nitrite. Appl Environ Microbiol 84(17):e01255-18.
